# Inflammatory Architecture of Frailty in the Realm of Alzheimer's Disease and Related Dementias

**DOI:** 10.1002/alz.086499

**Published:** 2025-01-09

**Authors:** Jiachen Chen, Margaret F Doyle, Yumeng Cao, Ahmed Ragab, Joanne M Murabito, Kathryn L. Lunetta

**Affiliations:** ^1^ Department of Biostatistics, Boston University School of Public Health, Boston, MA USA; ^2^ University of Vermont, Colchester, VT USA; ^3^ Boston University School of Medicine, Boston, MA USA

## Abstract

**Background:**

As a risk factor for Alzheimer's disease and related dementias (ADRD) in older adults, inflammatory mechanisms underlying physical frailty remain incompletely elucidated. This study aimed to characterize the inflammatory architecture of frailty and explore predictive implications of inflammatory signatures of frailty on ADRD.

**Method:**

The study included 741 Framingham Heart Study Offspring cohort participants (52% female, mean 60 years range 40 to 85), dementia‐free at Exam 7 (1998‐2001), followed for incident dementia over 15.8 years on average. The sample was randomly split 50/50 into discovery and validation sets. A total of 209 circulating inflammatory markers including peripheral immune cells and inflammatory biomarkers were measured at Exam 7, with Fried frailty index assessed at Exam 8 (2005‐2008). Using frailty and its 5 components as outcomes, LASSO was conducted on inflammatory markers adjusted for covariates in discovery set. Inflammatory signatures were generated by summing the product of regression coefficients and marker levels for each participant. Pairwise associations between 6 signatures and incident dementia/AD were conducted by Cox models in validation set, with FDR ≤0.05 declaring significance.

**Result:**

A total of 48 inflammatory markers were involved in signatures: 9 protein biomarkers (4E‐BP1, ADA, CCL3, CD40, CDCP1, HGF, IL‐10RB, IL‐12B, and IL‐17C) and 4 immune cells (CD4 T helper cells, CD4+FoxP3+, CD8+FoxP3+, and TREM2+ NCM) contributed to at least two signatures. In the validation set a handgrip‐strength‐specific signature was significantly associated with incident dementia (HR=0.50, 95%CI: (0.32, 0.76), FDR=0.01) and incident AD (HR=0.44, 95%CI: (0.25, 0.76), FDR=0.01), a walking speed‐specific signature was associated with incident dementia (HR=14.30, 95%CI: (3.67, 55.73), FDR=<0.01) and incident AD (HR=13.95, 95%CI: (2.65, 73.49), FDR=0.01). An unintended weight loss‐specific signature was significantly associated with incident dementia (HR=3.45, 95%CI: (1.09, 10.94), FDR=0.05), and exhaustion‐specific signature was associated with incident dementia (HR=4.12, 95%CI: (1.59, 10.69), FDR=0.01) and incident AD (HR=5.73, 95%CI: (1.84, 17.90), FDR=0.01), and overall frailty signature was associated with incident dementia (HR=2.33, 95%CI: (1.13, 4.84), FDR=0.04) and incident AD (HR=2.82, 95%CI: (1.08, 7.37), FDR=0.05).

**Conclusion:**

Frailty‐implicated signatures unveil inflammatory markers related to the biology of frailty and ADRD. Future replication studies are needed to inform ADRD prediction signatures.

